# Advancing Global Alzheimer's Disease Research: Insights from the Catholic Brain Health Center's Cohort and Innovations in Biomarker Development

**DOI:** 10.1002/alz70856_100283

**Published:** 2025-12-25

**Authors:** Hyun Kook Lim

**Affiliations:** ^1^ The Catholic University of Korea, Seoul, SEOUL, Korea, Republic of (South)

## Abstract

**Background:**

The Catholic Brain Health Center in South Korea manages one of the region's largest Alzheimer's disease (AD) cohorts, encompassing over 6,200 cross‐sectional and 1,400 longitudinal datasets. Since 2017, the center has utilized advanced imaging modalities, including amyloid and tau PET scans, to investigate regional variations in AD pathophysiology, risk factors, and therapeutic responses. These efforts provide critical insights into biomarker development and patient stratification, enabling the identification of high‐risk populations and optimization of clinical interventions.

**Methods:**

The center integrates clinical, neuroimaging, and biomarker data to develop predictive models and facilitate early diagnosis and monitoring of disease progression. Global collaborations, such as with the DAVOS Alzheimer's Collaborative, ensure methodological rigor and scalability in harmonizing local research with international frameworks. Plans to share this dataset via platforms like the Alzheimer's Disease Data Initiative (ADDI) are underway, promoting equitable access and global data harmonization.

**Results:**

Significant advancements have been made in identifying biomarker‐driven diagnostic approaches. Predictive models combining cognitive and neuroimaging data demonstrated high accuracy in early AD detection and monitoring disease progression. Regional analyses have highlighted distinct AD pathophysiological patterns, contributing to personalized therapeutic strategies. Collaborative efforts have accelerated data harmonization, improving the scalability and precision of research outputs.

**Conclusions:**

The Catholic Brain Health Center exemplifies the transformative potential of integrating localized research with global initiatives. By focusing on harmonized data sharing, predictive analytics, and addressing regional disparities, the center is setting a foundation for impactful advancements in Alzheimer's disease research. Future initiatives aim to enhance research equity and facilitate the global application of biomarker‐driven diagnostics, fostering innovation in early detection and intervention strategies.

